# Fort Donelson

**Published:** 1862-04

**Authors:** 


### FORT DONELSON.

Your correspondent was one of the favored twenty selected by the Sanitary Committee of Chicago to go to look after the wants of our wounded at the fight at Fort Donelson. Tuesday morning found us aboard the train on the *Illinois Central* bound for Cairo, with hospital stores and delicacies. The Board of Trade and Citizens' Committees were also along, which gave us quite a deputation from Chicago.

Our train was hailed by anxious crowds at every depot, the fact of Donelson's surrender being rumored at most of the points we came to, and the shouts that went up from the assembled hundreds told of the response it met in their feelings. We had constant accessions, until sitting room was at a premium—friends starting to look after sons, brothers, and there was an air of anxious contentment on most countenances, that few could look on unmoved. They seemed to suppose their friends dead, but the cause in which they died dissipated every other feeling. One mother going to look after her only son, especially attracted my attention, as she would mingle her hopes and fears in her conversation, it was easy to see which a mother's anxieties placed foremost.

As night approached, thoughts of sleeping cars were suggested, and Mr. Wicker, our energetic spokesman, telegraphed to Odin to ascertain the chance of securing one for our delegation. It was announced secured, and the illusion was only dissipated upon arriving at Odin, finding every place taken, the cross trains, on similar errands, having secured every birth. A few of our party, by some exchange or courtesy, secured places, and I, by the kindness of Dr. Evans, was favored with a few hours rest. I did not sleep—as I never do—nor could I, had I been most amiably disposed to. There was such a constant uproar of snoring as to drown the rattle of the cars. We had all varieties, from the high-toned amateur, to the old, well-developed, full bass. Some snored races, others at a mark; some by note, while others seemed, by their deliberate, dry, stiff getting-it-out, as tho' trying to spell it.

Our train was so long and heavy after leaving Odin, that before arriving at Cairo on an up grade—the track was slippery from the falling rain and sleet—we had three engines to take us along, and then had to take the advantage of down one hill to up the other.

We did not arrive at Cairo until 10 A. M. Wednesday, six hours behind time. Those who have been in that attractive and romantic locality, will appreciate the landing there, while it was pouring torrents, and the streets in their most hospitable mood. Their disposition to take you in was only equalled by their determination not to let you go, in so far we felt we were nearing Dixie.

I met Prof. Weber, of Cleveland, at Odin, who, as Surgeon General of Ohio, was going to look after the wants of the Ohio troops. We set out together at Cairo to find "Head Quarters," as only by passes can you get about in that martial law town. We found, as had been intimated before arriving, that no passes were issued to Donelson—orders absolute. The excuse given by Gen. Paine, was that his jurisdiction only extended to Paducah, and of course he couldn't. We took our passes to Paducah. Meantime, on learning of the determination not to allow us to go to Donelson, our Chicago delegation had appointed Dr. Evans and Judge Scates to wait on Gen. Paine and inform him of our wishes, and urge an exception to the rule—which was so far successful, after very much trouble and urging, that while he did not issue passes himself, Dr. Taggart was furnished with blanks, and those desiring one marked for "Donelson," among which I was numbered.

When this point was gained, the next anxiety was to get a chance to go, as the running of steamboats was a matter of no certainty. However, we got word that the Governors of Indiana and Illinois, with their staffs, were on a Government boat, "Belle of Memphis," going up to Fort Donelson—and *had been for twenty-four hours.*

That was suggested as the most certain way of getting up, and so we got our baggage aboard, where we found a number of our delegation. The boat was very much crowded, the state-rooms all taken by the "staffs" and Governors, and, from what I heard of one of the latter officials, he found it necessary to keep his closely. He got so discouraged at the delay, that it depressed him.

Within an hour of starting and near night, our passes were called in question, and were decided of no use—could not go on them, not being properly countersigned. Word came from another boat, the *John Warner*, where the balance of our friends had taken passage, that a similar trouble existed there, and it was pay, or *go*, and pay *if* you go. Col. Cummings being aboard, interfered and used his best influence to get our matters straight, offering to certify to our mission, etc. The amiable clerk at last agreed to carry us to Donelson, but must lift our passes, so that we could get back as best we might—unless we came back when he did. It was finally concluded to risk the getting back, and we remained on board.

The whole was gotten up for effect, for, as we ascertained afterward, the boat being employed by the Government at so much a day, our passes were good and he was not required to have any evidence of having carried us—to obtain his pay, as he asserted—as also further proven by the fact that our passes *were never asked for.*

The embarrassment at Cairo had a very unfavorable influence on many of our company, who seemed to be no friends in the first instance to "red tape." It is not according to our go-ahead way of doing things—a man's mission being all the pass he needs. Not so there, everything went according to rule, greatly to our annoyance. All well no doubt, and yet it was rather discouraging for men who left profitable professional employment many of them, and others occupations not less so, to attend to the wants of sick and dying men, to find men standing resolutely in the way of its accomplishment. The impression left, though the rule may be right, was not good.

Our steamboat troubles, we found were not so much owing to the trouble about passes as to the Captain's political views. He was a Secessionist, whose boat had been impressed.

In the afternoon, while waiting, I visited the gun boats. They are formidable looking machines; sides slanting from the water to the top, or hurricane deck of ordinary boats, upon which the pilot house is situated, whose sides also slant. The sides are iron-plated, which forms a uniform covering, except the port holes for the pilot to see through. These slanting sides give the striking ball, an obliquity which increases their aptitude to glance. The smoke stacks, with the pilot house, are the only objects which interrupt the uniformity of the vessel, except a narrow walk around the outside, on a line with the port holes. I first visited the *Tyler*, upon which I had the good fortune to meet my friend Dr. Jones, of Cincinnati, who was its Surgeon. He showed me in detail its inner works. The inside is kept scrupulously clean. The guns, of various size and beautiful construction, seem to be the most perfect of all war apparatus. They are kept in perfect order; vary in number—four on the sides and three at each end, being, I think, the usual number. The *Tyler* is entirely new, never having been in service. These gun boats seem altogether better fitted for serving cannon than any other. Everything can be kept in its place; no dragging them about on land to get stuck in the mud; men as well protected as possible; port holes just large enough to allow the men to stand beside while they load. But the shock must be tremendous in the confined room. Wonder if they have had the suggestion, of glycerine on cotton to fill the ears, made to them? I forgot it.

I next visited the *Essex*, where I found another friend, Dr. Rice, as Surgeon, formerly of this city. Capt. Porter is Commander. One needn't go very far over that boat to see that it has seen service. It was at Ft. Henry and received two balls, which went through it from end to end, besides numerous others. The front part of the boat is not yet plated, though it is painted black and from a distance it would be impossible to tell that it was not, but the enemy knew it. One ball struck directly in the forward end and went the entire length of the boat, hurting nothing except tearing the boards handsomely and coming out of the stern, which was also unplated. Another ball was more fortunate. It passed through one of the forward port holes, took off the hinder half of the head of Capt. Porter's aid, Brittain, and struck the steam pipe, cutting it, filling the room with steam, which went up filling the pilot house, scalding two pilots to death instantly and some marines. Captain Porter, as our readers recollect, was very severely scalded, but told me he had presence of mind to cram his scarf in his mouth. One of the sailors picked him up, and though he is a man weighing over two hundred, carried him on the narrow walk outside (9 inches wide) the entire length of the vessel, from before aft, during the progress of the battle, and so saved his life. The Captain related to me the attachment of one of our marines to our flag. One of these worst scalded, when it was announced that the white flag was up at Henry, raised himself up on his elbow—"Three cheers for the Stars and Stripes," said he, and, falling over, died with the words on his lips. The Captain was very severely scalded and his life was despaired of for several days. When I saw him his face was still scabbed over, and his eyes closed almost entirely, but he didn't talk as though there was, or even had been, anything the matter. He is decidedly a bold, fearless Commander, and a fit one to follow where our brave, pious old Commodore leads. I there met Lieut. Paulding, another, who, as well as Porter, are descendants of Commodores in the U. S. navy, whose names they bear, and whose history warrants us in expecting good things of them.

We got off up the river about 5 o'clock in the afternoon. Our accommodations were none of the best, but I secured what I could of a mattrass, only six feet in length, by the kindness of some friends, and, having slept none the previous night, enjoyed it finely. As to eating, we could get nothing. The steward pretended to serve those who wanted it at the inevitable four shillings, but, hungry as I was, I had no stomach for it. Like some of our single handed eating houses one pot of vegetables would last an entire season. How or by what process they alter the taste of their cooked food so that one cannot tell what it is, is more than I can imagine. Everything had a "gulchy" taste.

We had on board, that I recollect, Gov. Morton, of Indiana; Gov. Yates, of Illinois; Prof. Weber, of Cleveland; Dr. Douglass, of *Am. Med. Monthly*, who is connected with San. Commission; O. M. Hatch, our Sec. of State; ex-Gov. Wood, of our State; Comp. Ward, and Rev'ds Collyer and Tuttle, Drs. Rogers, Walker, Hahn, Andrews, of our city; Dr. Cornyn, of Gen's Lyons' staff.

We had rather a pleasant time, though we began to see the evidences of approach to our destination.

A gentleman got on at Paducah, who learned from one on board that his only son was killed. It could not fail to move the heart of any one to see the tears rnnning down the sunburnt face of that devoted father, while he told of that only, dear boy, before he left home last spring, planting the roses over and adorning the grave of an only daughter.

We arrived in sight of Donelson on next afternoon, Thursday. The afternoon was very chilly, though all were on deck, and, as we neared it, one thing was certain—the admirable selection for defence. The Fort is situated on an eminence or bluff; with its water batteries below, commands the river, which approaches it—a straight line for three miles—with no way to come except straight up to it, unprotected by the slightest bend in the river. The banks bore evidence of the recent conflict.

When we arrived at the landing, I got my pass, and having friends in the gallant Iowa Second, parted with Prof. Weber, expecting to meet him on the morrow, and, by the way, we haven't met yet.

It was getting dusk before I set out on my uncertain errand of finding a regiment among forty, without any directions as to where they were or how to find out. I started over the hill to get among the camps to get my information if possible. I got over the hill, and into the mud, and dark and brush, and stumps and water, and hollows and ravines, and creeks and logs, and every place except where any one could tell me anything I wanted to know. You can get a good idea of their knowledge of each other's regiments by the knowledge of individuals of each other in large citizes.

Nothing daunted, still I went. Some one suggested that, as they had fought on the right, they *might* be in the Fort. That was hint enough to walk a few miles on at least, and off I started at the point of a man's finger for the Fort. To undertake to describe how I got to that Fort that night would tire the patience, if it didn't the credulty, of your readers; how I came to bottomless roads, necessary to cross, and yet found the *bottom;* impassable creeks, and yet *passed* them; changed guides twice, both picked up by chance, and yet the very men most serviceable; and when at last I got into the Fort, found our regiment there, and, above all, my friends both safe, I may leave my readers to imagine that I felt as though rescued.

The next morning (Friday) I went, in company with Col. and Adj. Tutle, Surgeon Marsh and Assistant Surg. Nassau, to view the scene of the late conflict, and their glory. To describe everything I saw would require more time than I can spare, and perhaps ere now my readers are beginning to think this a curious professional letter. I didn't have the least idea when I began of writing a professional letter—only things as seen by a professional man.

The Iowa Second Regiment is the regiment of honor, as having led the charge on the intrenchments and being the first inside. I saw the place of their charge and entrance, and I must say it was an accomplishment worthy of the praise it has received. All honor to the Surgeons of the regiment, for they did their whole duty. The Surgeon established his quarters, and the Assistant Surgeon, Dr. Nassau, followed with his squad between the wings within fifty paces of the entrenchments, where his men began to fall, and carried them to the rear. There was no deputy needed for him; he went himself. For cool courage and successful accomplishment that charge has few parallels in history, and received the merited compliments of the rebels themselves.

Several narrow escapes by the Colonel were told me. A cannon ball struck a log on which he was standing, knocking it from under him, injuring him by the fall very severly. While he was crossing the head of a ravine he saw two men lying by the root of a tree, both aiming at him, not more than thirty paces distant—both missing. The bullets passed by his head, when one of his privates stepped forward, fired, and killed both of them. A ball passed through his gauntlet, striking the hilt of his sword, and paralizing his hand for a time. The regiment suffered much. Out of about 500 men taken into battle, 202 were either killed or wounded.

We followed around the entire line of entrenchments. The hardest fighting was done on the extreme right. Everything betrayed the sanguinary struggle that had there taken place. Horses dead; filled trenches marking the graves of the slain; some marked by boards with names and regiment; others without any mark; trees cut off, barked and scarred; hats with holes through and blood marks inside; cartridge boxes, canteens, and everything in mingled confusion, with here and there the hitherto undiscovered remains of some victim. At one point at that part of the entrenchments I noticed that the trees and underbrush were unusually marked by bullets. I saw, in passing a hundred yards, not a single one untouched. One tree six inches across had in the first eight feet of it fifteen bullet marks.

From the right of the entrenchments we passed through the little town of Dover to the river. In passing I called at a single hospital, which was mainly occupied by rebel wounded. The Surgeon was a Dr. Metcalf, of this State, and a rebel Surgeon, Dr. Holcom, of Miss. The patients seemed to be well cared for and in good condition. The hospitals of Dover are the larger dwellings, churches, while others are placed in sheds, and many of them in most destitute condition. I saw none of these myself, but had it from others that they were most deplorably cared for, lacking everything that could assuage the sufferings of the wounded or give them a chance for life. Their surgical attendance is entirely inadequate, and there is no effort made by the inhabitants, though the wounded there are nearly all secessionists, to give them relief. Their Surgeons were allowed to attend their wounded, and, when told of their needs, quietly said they had nothing to do with them when they did not belong to their regiments.

I arrived at the river about 2 P. M., and set out about finding where there was work to be done. I went aboard the *City of Memphis*, and found it to be the Hospital Boat, and without a Surgeon except the boat Surgeon, Dr. Turner himself. This boat receives the sick and wounded as a depot, and then sends them off by other boats, as may be directed. There were on board over 200 wounded soldiers, and one may well wonder how much headway one Surgeon could make in such a list of cases, and in addition have the entire charge of the boat, moving patients, &c.

It is said that the amiability and professional courtesy of the polite and well disposed physicians of a neighboring city are chargeable with this delightful and humane state of affairs; but of this I know nothing more than rumor, and hope for the sake and in the name of everything honorable it was not so.

I met Dr. Turner, who politely invited me to go work, and I was soon engaged in all the military surgery the most anxious could desire. When I started I began alone to work on one end of the long tier of cases. Very soon Prof. Andrews, of this city, came aboard, and we divided the boat between us. Soon, also, Drs. Rogers, Hahn, Walker, of this city, Dr. Perry, of Hunter Station, and Dr. Wyman, of Rockford, came and gave us valuable assistance.

The wounded occupied the cabins of the boat from one end to the other; arranged with their heads towards the state rooms, and as close together as their mattrasses could be placed. The state rooms were all filled also, except a few at the stern for the officers and nurses. The sick were placed principally in the state rooms, the wounded outside.

Nurses were detailed from the regiments there to wait on the sick, and some were most valuable, while some of them were indifferent, and the first night many of them went to bed and left the volunteer help to provide for their wants.

We had some most serviceable help of that sort from this city, and some whose aid was invaluable, and such as should entitle them to the everlasting gratitude of Chicago. I particularly mention Comptroller Ward, A. G. Downs, Rev'ds Collyer and Tuttle, and Mr. Munson. These gentlemen did a noble and devoted part by these poor wounded, suffering and dying men. Many a look of unspoken gratitude was given to them from the fast glazing eye of the dying soldier.

We had a work before us that could well move the deepest feelings of the most insensible heart. There lay twelve score of men who had perilled their lives for us and our country. The misfortunes of war in their worst shape had overtaken them, and quiet, gentle, comforting words of all, both Surgeons and nurses, tended as far as could be to make their sufferings bearable, and showed how profoundly sensible they were of the character of the cases they had in charge. I continued up, working with my cases until three o'clock next morning, and by that time we had succeeded in redressing all the wounds and making them comparatively comfortable.

The Surgeons who had previously dressed the wounds seemed impressed with the idea that the quantity was of much importance, and as a consequence bullet wounds were well linted and closely bandaged over to prevent the escape of matter. The trouble seemed to be *too much* dressing. One case of gun-shot wound of hips must have had three full length rollers on it, or near thirty yards.

The simplest possible dressing was used, and where it could be applied, nothing more than a compress dipped in cold water, and frequently changed. One Surgeon is mentioned at Paducah, who, from want of anything else, or his own originality, dressed his wounds with blistering cerate.

The wounds were of almost every possible variety, and I call up from memory, after the lapse of two weeks, some of those that I saw.

Two wounds of head by balls, both balls entering brain; one had died and other would. One struck by passing ball, depressing skull; I trephined him, but he would probably die. One upper jaw shot out; one lower jaw struck by a ball at symphysis and broken into four pieces, one piece taken out and others very much dislocated by action of muscles. He was a brother of one of our students of 1860–1, who was with him and giving him a brother's care. Erysipelas also broke out in his face; treated with Iron locally and Iron and Quin. internally. One case a bullet entered the external ear coming out on the opposite side between the jaws in front of the ramus, cutting the palate severely. One case a bullet passed between the esophagus and trachea. One a bullet entered as the body was stooping at the lower end of the scapula coming out on the neck in front, cutting the apex of the lung. Three cases of bullets directly through lung, all died. Two were of Minnie balls—holes very large—and both of them suffered very much. Several wounds of the arm and forearm; one a fracture of the surgcial neck by a ball. One ball struck a man lying on his side in the epigastric region and following between the layers of muscles came out on the back part of the hip. One, the son of the lady I mentioned as going to look after her son, was shot directly through the right illiac region, the ball remaining in, and he well enough to be removed home—no seeming trouble. One poor boy shot directly through the sacrum, suffered terribly, paralized bladder, though his bowels moved naturally. Had also another wound through thigh.

Very many were shot through lower limbs; three or four through knee-joint; two through ankle-joint. Those through the joints, as inflammation arose, suffered intensely. We had several cases of amputation on board which had been performed. All the operative surgery had been performed before, as it was a week after the battle. The wounds made by the balls of different kinds were generally easily distinguishable. The modern minie ball, from its great size and tearing qualities, will most likely largely increase the mortality from wounds, of those not killed immediately. The inflammation in joints arising from such balls, must need be destructive.

The wounded were mostly very patient and easily satisfied, and grateful for attentions. They seldom complained, and with few exceptions, and these with every reason, made but little noise. One poor fellow, whose breast had been bored through by a Minnie, suffered excruciatingly at every breath—seemed as though he couldn't die. I think I never had my sympathies more completely stirred. His name was Allen, from

Effingham, in this State. I raised him often, to try to cough the mucus and blood from his clogged throat, and the air would escape by the bullet holes, frustrating his object, until I stopped them with my fingers.

One soldier named Medland—31st Illinois, Co. B—was in the first instance shot through the knee-joint. He did not give up, but still kept firing. Another ball struck and went the whole length of the thigh—a flesh wound; and still he fired until a ball coming shattered his left arm and stopped his handling his gun. This man then lay on the field 2½ days, without food, drink, or shelter, before his wounds were dressed. I had no more resolute man, or more cheerful one, under my care, and he talked happily of the time when he would be well enough to try it again.

Another almost similar instance of a young man named Broderick, 8th Illinois, first shot through hips, then arms, and not until a ball broke his gun, shattered his left hand, did he desist.

Surgeon Marsh told me that of his two hundred and two wounded and killed, but a single man moaned, and he was shot through the lungs.

On Friday afternoon we changed to another boat seventy-five of our cases—bound for Cincinnati. On Saturday morning we had some stores from Chicago put aboard: wines, jellies, lemons, brandies, and it was a great comfort to our suffering patients. The suddenness with which such a number of wounded men are precipitated on us must be some apology, and yet there must have been criminal negligence somewhere, for our boat was unable to obtain a pound of fresh meat to give our patients, and instead of animal broths we had to feed them on indifferent corn gruel. I maintain that when men are willing to peril their lives for us, the least we can do is to take good care of them, and do it promptly. We left on Saturday morning and arrived at our destination, Mound City, the next morning.

We lost six or eight on the way. Two cases of amputation died; one of the leg and one of the thigh. I noticed particularly how excessively painful their wounds become as collapse set in; to me an unusual thing in other practice. It rained Saturday; and while we kept as well aired as possible, the stench become almost unsupportable. The consequence was we had four cases of Erysipelas before we got to Mound City.

I went through the hospital at Mound City on Sabbath, and was greatly pleased at the admirable arrangements it contains. It is one side of an entire square, upon which a business block of buildings had been erected. They have been fitted in a substantial and convenient manner for wards, and it makes a magnificent hospital. Everything about it is marked by order and care for the inmates. The building is three stories high, each of which furnishes a ward the size of the intended store below. The wards are very numerous, well ventilated, light, clean and comfortable. The patients and their beds are clean. Their food is of good quality, well cooked, and put before them in an eatable shape. Many of them I noticed with articles of luxury that must have been gratifying to their dainty palates.

I saw of the Sisters of the Sacred Heart there, who have the supervisory care of the nursing, and admirably is it done. I was more than pleased at the quiet, orderly and unobtrusive way in which they performed their deeds of nursing. I cannot say so much, however, of the generality of our volunteer female nurses. I think they are a nuisance, and one that ought to be abated instanter. It is the opinion of the Army Surgeons also. Many of them are very excellent women, but such as are, seldom retain their excellencies. I think a female, to go into the wards of an hospital as a common nurse, is sadly out of place; and if she has not already lost her womanly delicacy, will not be long in doing so, and either be slandered or *not*. Besides, very few women with minds right, their hearts and everything else in the right place, propose themselves for such a place. But there are services that can be done by none so well as by women: the preparation of food, care of the linen, and cleanliness of patients, preparation and administration of delicacies. These, with other nameless hundreds of kind offices, can be done by none so well as women.

I found the wards there supplied mainly by volunteer Surgeons, and the care, skill and humanity showed by them in their duties is worthy of all praise. I met there Dr. Rooker, of Indianapolis, Dr. Riddich, student of Rush Med. College, Drs. Winchester and Adams, of Elgin, Dr. Fishbach, of Indiana, gentlemen whose qualifications give ample guarantee for the service they render.

The only unpleasant fact in connection with this hospital is that it is under the charge of a homœopath. But, as none of my acquaintances seemed to know it who were attending there, I don't suppose he intrudes either himself or opinions on them.

I append, by consent, Prof. Andrews' list of location of injuries, taken from *Medical Examiner*:

Wounds of cranium, 14; of scalp, 19; of eye, 4; of jaw, 4; of chin, 2; of tongue, 1; of ear, 3; of mouth, 4; other parts of face, 10; of neck, 8; fractures of shoulder, 13; flesh wounds of shoulder, 30; fractures of arm, 16; flesh wounds of arm, 27; wounds of elbow, 4; fractures of forearm, 4; flesh wound of forearm, 4; fractures of hand, 25; flesh wound of hand, 11; wound of chest penetrating cavity, 10; wound of chest not penetrating cavity, 10; wounds of back, 5; wounds of abdomen, 7; fractures of hip, 7; flesh wounds of hip, 8; fractures of thigh, 9; flesh wounds of thigh, 37; fractures of knee, 2; flesh wounds of knee, 7; fractures of leg, 9; flesh wound of leg, 27; fractures of foot, 4; flesh wounds of foot, 2; powder burns, 2. Total, 360.

